# Effect of intraoperative music on quality of recovery after arthroscopic knee surgery: a prospective, double-blind, randomized controlled trial

**DOI:** 10.12701/jyms.2026.43.29

**Published:** 2026-04-09

**Authors:** Ji yong Yeom, Soomin Kim, Hyeon Seung Yi, Juhee Min, Eun Kyung Choi

**Affiliations:** 1 Department of Anesthesiology and Pain Medicine, School of Medicine, Kyungpook National University, Daegu, Korea; 2 Department of Anesthesiology and Pain Medicine, Yeungnam University College of Medicine, Daegu, Korea

**Keywords:** Arthroscopy, Knee, Music therapy, Postoperative period, Recovery of function

## Abstract

**Background:**

Various perioperative therapeutic strategies have been used to minimize postoperative complications and enhance recovery. Music intervention has attracted increasing attention as a safe, noninvasive, and cost-effective approach with potential benefits for patient-centered postoperative outcomes. Accordingly, this study investigated the effect of intraoperative music on postoperative recovery quality, as assessed using the Quality of Recovery-40 (QoR-40) questionnaire, as well as its potential analgesic and antiemetic effects in patients undergoing arthroscopic knee surgery.

**Methods:**

Eighty-two patients were enrolled and allocated to either the music or control group. In the music group, patient-selected tracks were delivered intraoperatively via headphones. Postoperative recovery quality was assessed using the QoR-40 at 24 hours. Pain was evaluated 30 minutes, 2 hours, and 24 hours after arrival in the post-anesthesia care unit. The incidence of postoperative nausea and vomiting (PONV) was recorded at all time points.

**Results:**

Although the total QoR-40 scores did not differ between the groups, the music group demonstrated significantly higher physical comfort scores (*p*=0.006). The incidence of PONV at 24 hours was lower in the music group (*p*=0.048), whereas the postoperative pain scores and rescue analgesic requirements showed no significant differences at any assessment point.

**Conclusion:**

In patients undergoing arthroscopic surgery, intraoperative music was associated with improvements in physical comfort and PONV, despite no significant difference in the total QoR-40 score.

## Introduction

Various pharmacological and nonpharmacological perioperative therapeutic strategies have been implemented to minimize postoperative complications and enhance surgical recovery. Among these, music intervention has demonstrated beneficial effects in reducing perioperative anxiety and distress, mitigating postoperative pain and nausea, and improving patient satisfaction.

Since the initial description of the usefulness of intraoperative music in 1914 [1], its effects have been investigated in emotional, psychological, and physiological contexts [2-4]. This intervention represents a safe, noninvasive, and inexpensive modality relative to pharmaceutical approaches, and can be conveniently delivered in medical settings. Several relevant subgroups, such as methods of music delivery, timing of intervention, selectivity, and types of music, have been examined to assess the effectiveness of music interventions from the perspective of postoperative care and quality of recovery [5,6].

This study investigated the effects of intraoperative music on the postoperative recovery of patients who underwent arthroscopic knee surgery. The primary outcome was the total Quality of Recovery-40 (QoR-40) score at 24 hours postoperatively, on which the sample size calculation was based. Secondary outcomes included the QoR-40 subdimension scores, postoperative pain intensity, rescue analgesic consumption, and incidence of postoperative nausea and vomiting.

## Methods

**Ethics statement:** This prospective, randomized, double-blind study was approved by the Institutional Review Board (IRB) of Yeungnam University Hospital (IRB No: YUMC 2019-09-053) and registered with the Clinical Research Information Service (KCT0007171). Written informed consent was obtained from all participants before enrollment.

In total, 82 patients aged 20 to 65 years with a physical status classified as I or II according to the American Society of Anesthesiologists framework were scheduled to undergo arthroscopic knee surgery under general anesthesia. Patients who underwent emergency surgery, received routine analgesic therapy, were disoriented, or presented with impaired hearing were excluded.

The participants were assigned by computer-generated randomization to either the music group, in which music was provided during surgery, or the control group, in which no music was provided. After arrival in the operating room, standard monitoring (e.g., electrocardiography, pulse oximetry, and noninvasive blood pressure measurements) was continuously performed. The depth of anesthesia was assessed using the bispectral index. Immediately before anesthesia induction, the patients in both groups wore identical headphones. Patients in the music group selected their preferred music and the volume was adjusted to a comfortable level. The patients in the control group wore the same headphones without audio output. Anesthesia was induced with propofol (1.5–2 mg/kg) and remifentanil (0.05–0.2 μg/kg/min); rocuronium (0.8 mg/kg) was administered to facilitate endotracheal intubation. After intubation, music was delivered to the patients in the music group by an independent investigator until completion of the surgical site dressing; no audio intervention was provided to the control group. Anesthesia was maintained with 2 to 3 vol% sevoflurane in 50% oxygen with air and continuous remifentanil infusion at 0.05 to 0.2 μg/kg/min. During surgery, heart rate and blood pressure were maintained within 20% of preinduction values, and bispectral index values were maintained between 40 and 60. At the end of surgery, sevoflurane and remifentanil were discontinued. Neuromuscular blockade was reversed with intravenous pyridostigmine (0.2 mg/kg) and glycopyrrolate (0.01 mg/kg). To prevent postoperative nausea and vomiting (PONV), ramosetron (0.3 mg) was administered, and ketorolac (30 mg) was given to reduce postoperative pain. After endotracheal extubation, the patients were transferred to the post-anesthesia care unit.

Quality of postoperative recovery was evaluated using the QoR-40 questionnaire [7]. This instrument consists of 40 items across five dimensions: emotional state (nine items), physical comfort (12 items), psychological support (seven items), physical independence (five items), and pain (seven items). Each item was scored on a five-point Likert scale (for positive items, 1=none of the time and 5=all of the time; for negative items, the scoring was reversed), and the total QoR-40 score was calculated by summation. Total scores range from 40 to 200, indicating very poor to excellent postoperative recovery, respectively [8]. Patients completed the questionnaire 24 hours after being transferred to the post-anesthesia care unit.

A numerical rating scale (0–10) was used to assess postoperative pain at 30 minutes, 2 hours, and 24 hours after arrival in the post-anesthesia care unit. Patients with numerical rating scale pain scores higher than five or those requiring analgesic therapy were administered intravenous fentanyl (50 μg). The analgesic demand was recorded, and the incidence of PONV was evaluated at each time point. All assessments were performed by an independent investigator who was blinded to group allocation.

A difference of 10 points in the questionnaire score was considered 15% improvement [9]. Accordingly, the sample size was calculated to provide 90% power to detect a 10-point difference in QoR-40 scores, with an α error of 5%. In total, 37 patients were required in each group. Eighty-two patients were ultimately recruited to buffer against potential dropouts.

Statistical analyses were performed using IBM SPSS ver. 25.0 for Windows (IBM Corp., Armonk, NY, USA). Continuous variables are presented as mean±standard deviations, and categorical variables are presented as numbers of patients (%). Distribution normality was assessed using the Shapiro-Wilk test. The Student *t*-test was used to compare normally distributed continuous variables, and the Mann-Whitney U test was used to compare non-normally distributed continuous variables. The chi-square test or Fisher exact test was used for categorical variables, as appropriate. Statistical significance was set at *p*<0.05.

## Results

**Fig. 1.**
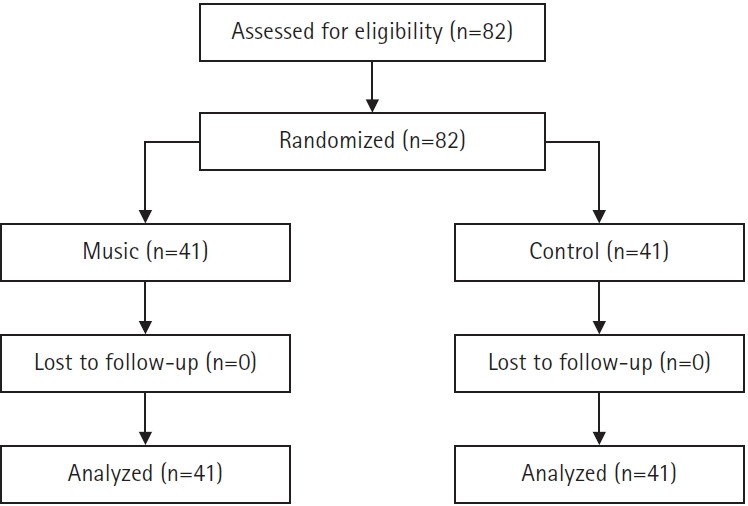
Consolidated Standards of Reporting Trials (CONSORT) flow diagram.

Among the 82 patients screened for eligibility, data from all the participants were analyzed (Fig. 1). The baseline demographic and perioperative characteristics, including age, sex, height, weight, duration of surgery, and duration of anesthesia, were comparable between the music and control groups (Table 1). Although no statistically significant difference was observed in overall QoR-40 scores at 24 hours postoperatively, patients in the music group demonstrated significantly higher scores in the physical comfort domain relative to patients in the control group (52.54±7.01 vs. 47.95±7.62, *p*=0.006) (Table 2). In contrast, the remaining QoR-40 domains, including emotional state, psychological support, physical independence, and pain, did not differ significantly between the groups (Table 2). The incidence of PONV at 24 hours was significantly lower in patients who received intraoperative music than in controls (two [4.88%] vs. nine [21.95%], *p*=0.048) (Table 3). However, postoperative pain scores measured using the numerical rating scale at 30 minutes, 2 hours, and 24 hours, as well as the frequency of rescue analgesic administration, showed no significant differences between the groups (Table 4).

## Discussion

In this study, intraoperative music exposure was associated with higher scores in the physical comfort dimension of the QoR-40 and a lower incidence of PONV 24 hours after arthroscopic knee surgery, while no significant effect on postoperative pain was observed.

Rapid return to daily activities is a key goal after surgery; therefore, effective control of postoperative adverse symptoms (e.g., pain, nausea, bowel dysfunction, and fatigue) is essential. Recently, increasing attention has been directed toward comprehensive functional recovery in postoperative care, encompassing emotional, physical, psychological, and pain-related domains. Numerous instruments have been developed to assess postoperative recovery quality, among which the QoR-40 questionnaire is one of the most widely used. This tool provides a multidimensional quantitative assessment of postoperative recovery and overall health status [10]. According to a systematic review by Gornall et al. [11], the QoR-40 demonstrates high validity, reliability, and clinical utility in the postoperative setting. To date, most studies have focused on the effects of music on preoperative anxiety, postoperative pain, and nausea; relatively few investigations have evaluated comprehensive recovery quality using the QoR-40.

The QoR-40 consists of five dimensions: emotional state, physical comfort, psychological support, physical independence, and pain. In the present study, the music intervention was associated with improved scores in the physical comfort domain, which included items related to PONV. In addition to the QoR-40 assessment, we independently evaluated the incidence of PONV during the first 24 hours, revealing a favorable effect of music intervention. Consistent with these findings, a recent meta-analysis demonstrated that music was effective in reducing PONV within the first 24 hours after surgery [12]. The authors of the meta-analysis suggested that PONV is influenced by anxiety, stress, and surgery-induced pain, all of which may be attenuated through music interventions, thus mitigating PONV [12]. Collectively, these observations suggest an association between intraoperative music exposure and reduced postoperative nausea in patients undergoing arthroscopic surgery, although the causal inference is limited. The concurrent improvement in the physical comfort domain and reduction in PONV should be interpreted with caution. As this domain includes items related to nausea and vomiting, the observed improvement may be partly attributable to the lower incidence of PONV. As individual item-level data were not retained, we were unable to determine the relative contribution of nausea and vomiting to the overall improvement in the physical comfort domain. Therefore, the observed benefit may primarily reflect the antiemetic effect of music rather than a general physical recovery enhancement. Although causality could not be confirmed, autonomic modulation by music may have contributed to symptom relief.

The positive effects of music intervention on postoperative recovery quality have been attributed to several potential mechanisms. Cognitive activities, such as listening to music, can modulate the perceptual experience and influence the perceived intensity of postoperative discomfort or pain. This is supported by the understanding that pain is an inherently multidimensional and subjective phenomenon shaped by physiological nociceptive pathways and psychological processes [2]. Another mechanism is related to the reduction in sympathetic activity secondary to decreased anxiety levels. Although anxiolysis is most pronounced when music intervention is administered preoperatively, several studies have demonstrated that intraoperative and postoperative music exposure can exert beneficial clinical effects [13]. Taken together, recognition of the biopsychosocial nature of surgical stress and pain has promoted multimodal strategies beyond pharmacological treatment alone; in this context, music may attenuate postoperative pain responses by enhancing pleasurable sensory input [5].

However, in the present study, the intraoperative music intervention did not improve postoperative pain control. This may be related to anesthetic depth-dependent suppression of auditory processing, as auditory perception and cortical responsiveness are markedly reduced during general anesthesia, particularly at bispectral index values of 40 to 60 [14]. Consistent with this finding, auditory evoked potentials are progressively suppressed with increasing anesthesia depth, indicating an elevated auditory perception threshold during unconsciousness [15]. Under such conditions, the pain-modulating effects of music may be attenuated. Additional contributing factors may include insufficient music volume, inadequate noise isolation in the operating room, or a relatively high pain threshold associated with arthroscopic procedures. Additionally, aspects related to music delivery, such as the method of administration, timing of intervention, selectivity, and specific type or genre of music, were not standardized, and these variables may have influenced the observed outcomes. Moreover, Hole et al. [5] reported that the analgesic benefit of music was less pronounced when administered during periods of unconsciousness, such as under general anesthesia, than when delivered in a conscious state. Collectively, these considerations suggest that the lack of a significant postoperative analgesic effect observed in the present study reflects a state-dependent limitation of auditory-mediated pain modulation, rather than the absence of an underlying biological mechanism.

This study had several limitations. First, the optimal music volume during general anesthesia remains uncertain, particularly in relation to the awake state. Although the explicit memory of auditory stimuli is suppressed under general anesthesia, auditory processing may still occur to some degree [16]. Pharmacological agents used for anesthesia may further elevate the threshold of auditory reflexes, potentially altering the perceptual or physiological responsiveness to sound [17]. In addition, despite efforts to minimize acoustic interference, ambient noise in the operating room could not be strictly controlled. Collectively, these factors indicate that the volume of music used in this protocol may have been insufficient to achieve an optimal therapeutic effect in patients undergoing arthroscopic knee surgery. Second, preoperative anxiety induces adverse physiological responses, including sympathetic activation, increased levels of circulating catecholamines, and increased pain sensitivity [18,19]. These responses may negatively influence perioperative outcomes and impede postoperative recovery. Anxiety has also been associated with postoperative nausea, delayed mobilization, and lower patient-reported recovery scores [20]. In the present study, preoperative anxiety was not assessed or controlled; thus, its potential confounding effects could not be determined. Future studies incorporating standardized preoperative anxiety measurements and appropriate adjustments are necessary to clarify the independent effects of music intervention. Third, although improvements were observed in the physical comfort domain, the study was designed and powered to evaluate domain-level and total QoR-40 scores. Item-level contributions could not be assessed, limiting the ability to determine whether the benefits extend beyond nausea and vomiting. Finally, detailed patient-related risk factors for PONV were not systematically collected, and the potential influence of baseline risk imbalances could not be excluded. Therefore, the observed association between intraoperative music and reduced PONV should be interpreted with caution.

In this study, intraoperative music intervention did not significantly improve the total QoR-40 score in patients undergoing arthroscopic knee surgery. Although improvements were observed in the physical comfort domain and PONV incidence, these findings may primarily reflect antiemetic effects rather than a broad enhancement of postoperative recovery quality. Furthermore, adequately powered trials incorporating symptom-specific analyses are required to clarify the overall effect of intraoperative music on postoperative recovery.

## Figures and Tables

**Table 1.** Demographics and baseline characteristics

Characteristic	Music group	Control group	*p*-value
No. of patients	41	41	
Age (yr)	41.2±14.2	39.8±13.8	0.649
Sex, male:female	27:14	22:19	0.368
Height (cm)	169.1±7.5	167.6±9.4	0.429
Weight (kg)	73.7±11.5	68.5±15.3	0.086
Operation time (min)	58.1±40.0	52.0±37.3	0.474
Anesthesia time (min)	93.3±43.5	87.7±41.9	0.553

Values are presented as number or mean±standard deviation.

**Table 2.** QoR-40 scores at 24 hours after the patient arrived at the post-anesthesia care unit

Variable	Music group (n=41)	Control group (n=41)	*p*-value
Emotional status	37.63±6.79	35.10±6.16	0.080
Physical comfort	52.54±7.01	47.95±7.62	0.006^a)^
Psychological support	31.98±4.47	31.76±3.08	0.797
Physical independence	19.44±4.77	18.98±5.16	0.674
Pain	27.17±6.28	28.51±4.36	0.265
Total QoR-40	168.76±22.88	162.29±21.81	0.194

Values are presented as mean±standard deviation. QoR-40, Quality of Recovery-40.

a) *p*<0.05, statistically significant.

**Table 3.** Incidence of postoperative nausea and vomiting

Postoperative time	Music group (n=41)	Control group (n=41)	*p*-value
30 min	3 (7.3)	5 (12.2)	0.712
2 hr	8 (19.5)	6 (14.6)	0.770
24 hr	2 (4.9)	9 (22.0)	0.048^a)^

Values are presented as number (%).

a) *p*<0.05, statistically significant.

**Table 4.** Postoperative pain and rescue analgesics

Variable	Music group (n=41)	Control group (n=41)	*p*-value
Numerical rating scale			
30 min	2.78±0.91	2.98±0.96	0.348
2 hr	2.10±0.77	2.24±0.80	0.401
24 hr	0.90±0.44	0.71±0.51	0.067
Rescue analgesics demand			
30 min	6 (14.6)	5 (12.2)	>0.999
2 hr	6 (14.6)	6 (14.6)	>0.999
24 hr	3 (7.3)	5 (12.2)	0.712

Values are presented as mean±standard deviation or number (%).
